# The current landscape of using direct inhibitors to target KRAS^G12C^-mutated NSCLC

**DOI:** 10.1186/s40164-023-00453-8

**Published:** 2023-11-04

**Authors:** Firas Batrash, Mahmoud Kutmah, Jun Zhang

**Affiliations:** 1 School of Medicine, University of Missouri Kansas City, Kansas City, MO 64108 USA; 2 Division of Medical Oncology, Department of Internal Medicine, University of Kansas Medical Center, Kansas City, KS 66160 USA; 3 Department of Cancer Biology, University of Kansas Medical Center, Kansas City, KS 66160 USA

## Abstract

Mutation in KRAS protooncogene represents one of the most common genetic alterations in NSCLC and has posed a great therapeutic challenge over the past ~ 40 years since its discovery. However, the pioneer work from Shokat’s lab in 2013 has led to a recent wave of direct KRAS^G12C^ inhibitors that utilize the switch II pocket identified. Notably, two of the inhibitors have recently received US FDA approval for their use in the treatment of KRAS^G12C^ mutant NSCLC. Despite this success, there remains the challenge of combating the resistance that cell lines, xenografts, and patients have exhibited while treated with KRAS^G12C^ inhibitors. This review discusses the varying mechanisms of resistance that limit long-lasting effective treatment of those direct inhibitors and highlights several novel therapeutic approaches including a new class of KRAS^G12C^ (ON) inhibitors, combinational therapies across the same and different pathways, and combination with immunotherapy/chemotherapy as possible solutions to the pressing question of adaptive resistance.

## Introduction to KRAS and G12C mutation in NSCLC

KRAS proto-oncogene, GTPase (KRAS), also known as Kirsten rat sarcoma viral oncogene homolog, has been a point of intense research since its discovery in malignant lung tissue nearly 40 years ago [1]. Comprising 33% of lung, 91% of pancreatic, and 42% of colorectal cancers, KRAS mutations directly contribute to one million deaths annually worldwide [2].

Functioning as a switch in the cell proliferation and survival signaling cascade, KRAS activation is reliant on the help of guanine nucleotide exchange factors (GEFs) which mediate the transition from the guanosine diphosphate (GDP)-bound (OFF) to the guanosine triphosphate (GTP)-bound (ON) [3]. Once ON (activated), KRAS then activates multiple downstream pathways including the RAF–MEK–MAPK and PI3K–AKT–mTOR pathways which stimulate cell growth and survival [3–8]. When the upstream signal ceases, KRAS relies on the GTPase activator protein (GAP) and its own intrinsic GTPase capabilities to hydrolyze the GTP back to GDP and return to the inactive, non-signaling state [3].

Under normal circumstances, KRAS is an integral part of cell function, however, once mutated, as is the case in 84% of all RAS mutations, KRAS can become the catalyst for cancerous growth, and has been explored particularly in non-small cell lung cancer (NSCLC), colorectal cancer, and pancreatic cancer [5, 9]. As observed in patients with NSCLC, the presence of this mutation is a marker of poorer prognosis compared to patients with wildtype (WT) KRAS [5, 10–12]. Missense mutations to KRAS result in alterations to the switch-II region of the molecule which impairs effector molecule (GAP) binding and causes an elevated percentage of active GTP bound KRAS [13]. This high rate of activation in mutant KRAS is driven by its picomolar affinity for GTP, the high GTP/GDP concentration ratio, and the aforementioned reduction in effector molecule interactions with KRAS that reduce its GTPase capabilities [14–16]. The constitutive activation of the downstream proliferation and survival pathways is regardless of the status of its upstream signaling from receptor tyrosine kinases (RTKs) such as epidermal growth factor receptor (EGFR) [13]. Missense mutations on KRAS are somewhat unique in comparison to other RAS homologs (NRAS and HRAS) in that the predominant mutation site occurs at codon 12 [14]. This codon is especially important when discussing NSCLC as it accounts for the large majority of KRAS mutations (~ 92%) [17, 18].

The most common mutations on the 12th codon include G12V, G12D, and the most common, G12C, a glycine-to-cysteine substitution accounting for ~ 59% of all G12 mutations [11, 17]. As previously mentioned, KRAS mutations are particularly present in lung cancers, accounting for 27% of metastatic lung adenocarcinomas (in a pool of 1655 patients); within that quarter of cases, 93% were smokers which in itself is a negative prognosis factor [10, 19]. G12C accounted for 39% of KRAS mutations compared to 18% each for G12D and G12V [10]. While G12C and G12V are more common in smokers, G12D is more common in non-smokers [20]. The G12C mutation is generally categorized as having a high affinity to RAF and a high intrinsic GTPase activity, while still being bound to GTP at a rate of approximately 75% [2]. This state of mutant KRAS is considered part of a dynamic cycle that favors GTP rather than constitutive activation, a factor that improves the response to inhibitors that target that high percentage [21, 22]. Although this article focuses on KRAS^G12C^ mutant NSCLC, the direct inhibitors and their combination therapies discussed here is potentially applicable to other KRAS^G12C^ mutated solid tumors.

## Direct KRAS^G12C^ Inhibitors for the treatment of NSCLC

### Early inhibition efforts

On what is otherwise a smooth and shallow surface that lacks allosteric regulation sites, KRAS was thought to be undruggable by direct inhibition and previous research attempts were focused on the inhibition of its synthesis, trafficking, interaction, downstream signaling, or post translational modification [21, 23–26]. After folding, KRAS undergoes several post translational modifications that assist in membrane localization—farnesylation, geranylgeranylation, and palmitoylation [17, 23]. Drugs targeting these enzymes, such as farnesyl transferase inhibitors (FTI), were at the center of early inhibition efforts, but were ultimately unsuccessful when used on solid tumors [23]. Efforts to combine lonafarnib (an FTI) with other drugs like paclitaxel and carboplatin proved ineffective as well, and the phase 3 trial of such combination was terminated [23].

Many attempts have been made to target the downstream pathways of KRAS, specifically, the MAPK and PI3K pathways [27, 28]. For example, through the inhibition of RAF association in cells, thus successfully reducing the phosphorylation of downstream molecules such as MEK and ERK, or direct targeting of MEK and ERK [24]. The small molecules developed such as selumetinib, which directly targets MEK showed early promise, however further research showed no statistically significant effects of the drug [24, 28]. Although targeting MAPK signaling downstream works effectively for some types of cancer and is indeed the standard of care for patients with BRAF^V600E^ activating mutations [29–31], such approach is proved to be largely ineffective in KRAS mutant cancers. In fact, this pathway was specifically tested on KRAS mutant NSCLC in the SELECT-1 study which showed that the addition of selumetinib to docetaxel did not improve either the median progression-free or overall survival in patients with previously treated KRAS mutant NSCLC [28]. Given the clinical ineffectiveness of drugs to either alter KRAS membrane localization or disrupt downstream pathways of KRAS, the only effective option is to inhibit KRAS directly.

While the biochemistry of KRAS was once seen as difficult to grasp given its complexity, recent breakthroughs in biochemical computational modeling and crystallography have allowed researchers to better grasp the structure of KRAS, despite its small size [24, 32]. Specifically, the ability to find small molecules to bind to the few binding sites that are specific to the given KRAS conformation has become a possibility. Going forward, the location of the glycine-cysteine substitution has been a key aspect of the research for a selective direct inhibitor of KRAS^G12C^ but also demonstrates one of the shortcomings of the current generation of inhibitors. The mutation was identified in a pocket (P2) beneath switch-II, a relatively novel structural discovery found on the GDP-bound KRAS by Ostrem et al. [33]. Thanks to KRAS^G12C^ having a cysteine residue so close to switch II, possible inhibitors of KRAS^G12C^ can use the thiol group of cysteine to form disulfide bonds, thus stabilizing the inhibitor at the switch site. However, this binding site is only accessible during KRAS inactivity when GDP is bound in the binding site as when GTP is bound to KRAS, the binding of the two forces a structural change, activating KRAS and changing the conformations of switch I and switch II [5]. In addition, the previously mentioned picomolar affinity for GTP makes accessing GTP-bound KRAS difficult [33]. Despite the challenges, this finding laid the groundwork for potential direct inhibitors on what was commonly termed an “undruggable” molecule [5].

#### ARS-853

Initial research by Shokat lab produced compound 12, the very first KRAS^G12C^ inhibitor. Despite the initial success of creating an inhibitor, further studies of the compound by Patricelli et al. found that the drug was unable to bind to KRAS in cells [34]. Soon after, Wellspring Biosciences created ARS-853, a structurally similar chemical that showed significant promise [35]. KRAS^G12C^ was inhibited by ARS-853, however studies by Lito et al. found that mutants that lack GTPase activity completely showed drastically reduced rates of ARS-853’s inhibition effectiveness [36]. Even when coupled with EGFR inhibitor erlotinib or MEK inhibitor trametinib, decreased levels of KRAS inhibition efficacy were noted [36], strengthening the notion that KRAS^G12C^ retains its intrinsic GTPase activity in vivo and loss of GTPase activity could be a potential mechanism of acquired resistance against inhibitors such as ARS-853.

#### ARS-1620

ARS-1620 is another direct inhibitor of KRAS^G12C^ that showed promise as the improved version of its precursor ARS-853 using an *S*-atropisomer possessing a quinazoline core that occupies the allosteric switch-II pocket (S-IIP) [37, 38]. According to Janes et al., it is more potent, selective, and more orally bioavailable (F > 60%) than its predecessor [39]. However, despite initial success in mice, use of ARS-1620 alone induced drug resistance to the KRAS-mutant tumor cells leading to short-term signaling adaptation or long-term selection of minor variants. To combat resistance, ARS-1620 can be used in triple combination with mTOR and IGF1R inhibitors to greatly increase its impact on KRAS^G12C^ both in vitro and in vivo [40].

### FDA approved inhibitors for treatment in advanced/metastatic NSCLC

#### AMG-510 (sotorasib/LUMAKRAS)

On May 18, 2021, the United States Food and Drug Administration (FDA) approved the first direct inhibitor of KRAS^G12C^ [41]. AMG-510, an *R*-atropisomer with a similar ligand structure to ARS-1620, is an irreversible inhibitor that specifically binds to the switch II pocket of GDP-bound KRAS^G12C^ utilizing a similar quinazolinone core to occupy the S-IIP and an acrylamide moiety to covalently bind cysteine-12 [38]. Produced by Amgen, AMG-510 proved to be both potent and effective [42]. In fact, it was the first small molecule inhibitor of KRAS^G12C^ to enter clinical trials (NCT03600883; Table 1). In comparison to ARS-1620, AMG-510 binds not only to the S-IIP region, but also utilizes an isopropyl substituent of the pyridyl ring to occupy the His95 groove on KRAS, allowing it to form 25 ligand–protein van der Waals contacts [35, 38]. These additional interactions enhance AMG-510’s potency approximately tenfold compared to ARS-1620 in a nucleotide-exchange assay with recombinant GDP-bound KRAS^G12C^ [35, 42]. Pharmacokinetic analysis of AMG-510’s phase I data showed that it has a half-life of 5.5 h with no dose limiting toxic effects observed [24]. A phase I/II study found that the most common adverse events of sotorasib included loss of appetite, diarrhea, fatigue, headache, cough, hot flashes, and nausea, along with severe adverse events in 6 patients, consisting of grade III pneumonia, malignant biliary obstruction, and grade IV pericardial effusion [43]. At the 960-mg once daily dose given to the NSCLC patients in the phase II study (NCT03600883, CodeBreaK 100; Table 1), patients experienced a median duration of response of 10.0 months and median progression free survival (PFS) of 6.8 months. AMG-510 showed grade 3 or 4 treatment adverse events (TRAEs) in 19.8% of patients with varying symptoms as mentioned earlier. This led to treatment discontinuation in 7.1% of patients and dose modification in 22.2% of patients [24, 44]. In the recent phase III study (NCT04303780, CodeBreaK 200; Table 3), sotorasib demonstrated an increased progression-free survival (5.6 vs 4.5 months) and a favorable safety profile compared with docetaxel; however, no overall survival benefit was observed [45]. The high drop-out rate in the docetaxel group due to the open-label design, as well as the fact that 34% of patients in the docetaxel group subsequently received a G12C inhibitor could be confounding factors [45].

#### MRTX849 (adagrasib/KRAZATI)

Another irreversible covalent inhibitor of KRAS^G12C^, adagrasib is another small molecule inhibitor similar to sotorasib (Table 1). With a longer half-life (24 h compared to 5.5 h for sotorasib) and extensive tissue distribution, adagrasib showed promise from its inception [46]. Adagrasib contains three subunits—*N-*methyl prolinol, chloronaphthyl, and substituted piperazine—attached to a tetrahydro-pyridopyrimidine core as well as a 2-fluoroacrylamide warhead on the distal side of the piperazine for covalent target protein binding [47]. Adagrasib was tested in a phase I/II KRYSTAL-1 study (NCT03785249; Table 1) in patients (N = 116) with KRAS^G12C^ mutation. The trial found that of the 112 patients with measurable disease at baseline, 48 (42.9%) had a confirmed objective response (median duration of response was 8.5 months) and the median progression-free survival was 6.5 months with an overall survival of 12.6 months as of January 15, 2022 [48]. TRAEs occurred in 97.4% of the patients with 44.8% were grade 3 or higher. GI TRAEs were predominant which occurred early in treatment and resolved quickly with successful management using dose interruption, reduction, and supportive care, resulting in only 6.9% discontinuation rate [48–50]. Despite being structurally similar to AMG-510, preclinical data has shown that adagrasib can penetrate into the central nervous system, specifically the brain and cerebrospinal fluid; moreover, clinical data has shown adagrasib has antitumor activity against brain metastases [51]. More recently, on December 12, 2022, the FDA granted accelerated approval to adagrasib for treatment of adult patients with advanced KRAS^G12C^-mutated NSCLC who progressed on 1st line standard-of-care, the same level of approval given to sotorasib [52].

**Table 1:** Comparison of AMG-510 and MRTX849 [11, 15, 17, 24, 25, 39, 41–43, 46, 48–50, 52–59]

Agent	AMG-510 (sotorasib)	MRTX849 (adagrasib)
Company	Amgen	Mirati therapeutics Inc
FDA approval date	May 18, 2021	December 12, 2022
Type of inhibitor	Irreversible covalent inhibitor of KRAS^G12C^	Irreversible covalent inhibitor of KRAS^G12C^
Mechanism of inhibition	Forms water bridges between the tyrosine residue found in the KRAS^G12C^ protein and the carboxyl group in sotorasib	Uses hydrogen mediated bonding of the hydroxyl group on the KRAS^G12C^ pocket with adagrasib’s pyrimidine ring
Number of NSCLC patients in phase I/II trial	N = 124 (NCT03600883)	N = 116 (NCT03785249)
Prior platinum-based chemotherapy and immunotherapy	Platinum based chemotherapy only: 11 (8.7%) Both therapies: 102 (81.0%)	Platinum based chemotherapy only: 2 (1.7%) Both therapies: 114 (98.3%)
Recommended starting dose based on phase I/II trial data	960 mg once daily	600 mg twice daily
Half-life	5.5 h	24.0 h
C_max_ of steady state (µg/ml)	7.5	3.25
Objective response rate (ORR)	37.1%	42.9%
Disease control rate (DCR)	80.6%	96%
Progression-free survival (PFS)	6.8 months (5.6 months in phase III)	6.5 months
Overall survival rate	12.5 months (10.6 months in phase III)	12.6 months
TRAEs occurring in > 5% of patients, all grades	Gastrointestinal toxicities, nausea, vomiting, elevated alanine transaminase (ALT) and aspartate aminotransferase (AST)	Gastrointestinal toxicities including nausea, vomiting, diarrhea; fatigue, increased AST and ALT, EKG QTc prolongation
Grade 3+ TRAEs in phase II trial	20.6%	45%
TRAEs leading to discontinuation	7.1%	6.9%
Preclinical data indicating ability to penetrate the brain and cerebrospinal fluid	n/a	Presence of adagrasib detected in brain and cerebrospinal fluid

### Other inhibitors entering clinical trial

#### LY3537982

An initial phase I trial (NCT04165031) of LY3499446, produced by Eli Lilly and Company, was terminated due to unexpected toxicity, however its successor, LY3537982 has begun to show promise. It has shown in preclinical studies to have a lower IC50 value than both AMG-510 and MRTX849 in addition to potent anti-tumor activity with complete regressions of KRAS^G12C^ tumors [35, 60]. In addition, it has been identified through mechanism-based combinatory screens that this drug has the potential for synergistic targeted therapies. These synergists include abemaciclib, LY3295668 (a selective aurora kinase A inhibitor), and cetuximab which when combined with LY3537982, showed both in vitro and in vivo enhancement of anti-tumor activity [35, 60]. A phase I clinical trial of LY3537982 monotherapy (50–200 mg BID, NCT04956640; Table 3) for patients with KRAS^G12C^-mutated cancer (n = 56) showed a promising safety profile with no recorded high-grade liver toxicity, an ORR of 60%, and a DCR of 80% [61]. In addition, it proved to be tolerable to patients who were previously intolerant to other KRAS^G12C^ inhibitors [61]. TEAEs observed in over 10% of patients were mostly grade 1, including diarrhea (38%), constipation (16%), fatigue (16%), peripheral edema (13%), and nausea (11%) [61]. Neutropenia was observed in one patient and no TRAEs or death were seen in this trial [61].

#### GDC-6036 (divarasib)

Created by Genentech and in an ongoing phase I clinical trial (NCT04449874; Tables 2 and 3) as both monotherapy and in combination with other anti-cancer therapies, GDC-6036 is being investigated for use in fighting advanced and metastatic solid KRAS^G12C^ tumors, including NSCLC [35]. GDC-6036 is a highly selective and potent KRAS^G12C^ covalent inhibitor that binds to the same switch II pocket as the other inhibitors mentioned, while irreversibly alkylating KRAS^G12C^ with more in vitro potency than sotorasib and adagrasib [62]. In the phase I trial using GDC-6036 as a single agent for previously treated locally advanced or metastatic KRAS^G12C^ mutant solid tumors, a recent report showed among 60 NSCLC patients, a confirmed ORR of 53.4% and a median progression-free survival of 13.1 months were observed [63]. TRAEs were observed in 127 out of a total 137 patients enrolled (including other solid tumor patients), with grade 3+ TRAEs occurred in 16 patients (~ 12%) and treatment discontinuation rate of 3% [63].

**Table 2:** Summary of Other KRAS^G12C^ inhibitors [35, 56, 59–61, 64, 66–69, 73–76]

Agent	Pharmaceutical Company	Clinical Trial (CT)	ORR of CT	DCR of CT	TRAEs
*LY3537982*	Eli Lilly and Company	Phase I (NCT04956640)	60%	80%	Diarrhea, constipation, fatigue, peripheral edema, nausea, neutropenia
*GDC-6036*	Genentech	Phase I (NCT04449874)	53.4%	–	Rash, diarrhea, nausea, vomiting, dry skin, and paronychia
*D-1553*	InvestisBio	Phase I/II (NCT04585035)	40.5%	91.9%	Elevated AST/ALT increased, diarrhea, hypertension, hypokalemia, increased total or conjugated bilirubin, hypothyroidism, and nausea
*HBI-2438*	Huyabio International	Phase I (NCT05485974)	–	–	–
*JDQ443*	Novartis	Phase Ib/II (NCT04699188)	41.7% 54.5% (RP2D of 200 mg BID)	–	Fatigue, edema, diarrhea, nausea, vomiting, and peripheral neuropathy
*JAB-21822*	Jacobio Pharma	Phase I/II (NCT05009329)	70% (400 & 800 mg QD groups)	100% (400 & 800 mg QD groups)	Anemia, total bilirubin increase, and proteinuria
HS-10370	Jiangsu Hansoh Pharmaceutical company	Phase I/II (NCT05367778)	–	–	–
*IBI-351 (GFH925)*	Innovent Biologics Inc	Phase I/II (NCT05005234)	61.2% 53.3% (RP2D of 600 mg BID)	92.5% 96.7% (RP2D of 600 mg BID)	Anemia, white blood cell count decreased, ALT increases, and pruritus
*BI-1823911*	Boehringer Ingelheim	Phase I (NCT04973163)	–	–	–
*JNJ-74699157*	Johnson & Johnson	Phase I (NCT04006301)	–	–	Increased blood creatinine phosphokinase (grade 3–4)

*RP2D* Recommended phase 2 dose

#### D-1553 (garsorasib)

D-1553, created by InvestisBio, is an orally bioavailable small molecule inhibitor of KRAS^G12C^ proven to be highly potent in vivo using cell line-derived xenograft tumor models. It demonstrated anti-tumor activity both as monotherapy and in combination with other targeted therapies. D-1553 selectively inhibits ERK phosphorylation in NCI-H358 cells harboring KRAS^G12C^ mutation. D-1553 exhibited tumor growth inhibition in lung cancer patient-derived xenograft models [57]. It is in a phase I/II trial (NCT04585035; Table 3) that is evaluating D-1553 both alone and in combination with other therapies in NSCLC and solid tumors in adults [35]. In the recently published phase 1 study among KRAS G12C mutant NSCLC patients, a confirmed ORR and disease control rate (DCR) of 40.5% and 91.9% respectively was reported. In addition, for patients with brain metastasis, ORR and DCR was found 17% and 100% respectively [64].

#### HBI-2438

Created by Huyabio International, HBI-2438 is an orally bioavailable inhibitor of KRAS^G12C^. It is currently recruiting for its phase I trial in which it will test its monotherapy efficacy with patients with lung cancer, colorectal cancer, and other solid cancers (NCT05485974).

#### JDQ443 (opnurasib)

Designed by Novartis, JDQ443 is a selective, covalent, and orally bioavailable KRAS^G12C^ inhibitor. JDQ443 is a stable atropisomer featuring a 5-methylpyrazole core and a spiro-azetidine linker designed to position the electrophilic acrylamide for optimal engagement with KRAS^G12C^. Its substituted indazole at pyrazole position 3 creates novel interactions with the binding pocket that does not include residue H95, allowing it to overcome resistance mutations that would otherwise prevent the efficacy of the drug [65]. JDQ443 is now in clinical development after its early phase data produced from an ongoing Phase Ib/II clinical trial (NCT04699188; Table 3) showed promise [65]. It has shown in this trial (n = 38) to have a 41.7% confirmed ORR at its recommended dose of 200 mg twice daily in its phase Ib study in patients with advanced NSCLC. TRAEs occurred in 71.4% of patients and included 4 grade 3 TRAEs but no grade 4/5 TRAEs were reported. The most common TRAEs included fatigue, edema, diarrhea, nausea, vomiting, and peripheral neuropathy. The grade 3 TRAEs included neutropenia, ALT and AST increase, and myalgia. Showing great promise, JDQ443 is also being tested in combination with TNO155 (SHP2 inhibitor) and/or tislelizumab (anti-PD-1 monoclonal antibody) [66, 67]. In addition, JDQ443 is currently undergoing a phase III trial to evaluate the efficacy and safety of JDQ443 monotherapy compared with docetaxel in KRAS^G12C^ mutant NSCLC patients who failed platinum-based chemotherapy and immune checkpoint inhibitor therapy (NCT05132075).

#### JAB-21822

Another oral inhibitor of KRAS^G12C^, JAB-21822, from Jacobio Pharma, is currently in an ongoing phase I/II clinical trial to assess its safety and tolerability both as a monotherapy and in combination with cetuximab in patients with G12C-mutated advanced solid tumors (EGFR inhibitor, NCT05009329). As of January 28th, 2022, the trial (n = 53) tested five different dose levels: 200 mg QD, 400 mg QD, 800 mg QD, 400 mg BID, and 400 mg TID. Current data shows no dosing-limiting toxicity observed along with identifying the common TRAEs which include anemia, total direct or indirect bilirubin increase, and proteinuria [68]. For patients with KRAS G12 mutant NSCLC on 400 mg QD and 800 mg QD, the ORR and DCR were 70% (7/10) and 100% (10/10), respectively, including 5 non-confirmed PR [68].

#### HS-10370

Developed by Jiangsu Hansoh Pharmaceutical company, HS-10370 is another oral small molecule inhibitor of KRAS^G12C^. It is currently beginning a phase I/II clinical trial (NCT05367778) to evaluate its safety and pharmacokinetics as a monotherapy. It is to be tested on advanced solid tumors but not primarily NSCLC. It is not yet recruiting.

#### IBI-351 (GFH925)

A covalent irreversible inhibitor of KRAS^G12C^, IBI-351 was designed by Innovent Biologics Inc. and is currently in a phase I/II trial (NCT05005234) to test its safety and tolerability as a monotherapy for advanced solid tumors harboring the KRAS^G12C^ mutation [56]. As of February 10, 2023, of the 67 evaluable KRAS G12C mutant NSCLC patients, 41 achieved PR, with an ORR of 61.2% and DCR of 92.5%. In addition, of the 30 patients treated at 600 mg BID (the recommended phase 2 dose), an investigator assessed an ORR 66.7% (confirmed ORR 53.3%) and DCR 96.7% were reported [69].

#### BI-1823911

Designed by Boehringer Ingelheim, BI-1823911 is claimed to be a more potent inhibitor than the two already FDA approved drugs for KRAS^G12C^ inhibition, sotorasib and adagrasib [70, 71]. It showed similar in vivo efficacy to the two drugs at 60 mg/kg of BI-1823911 compared to 100 mg/kg of either sotorasib or adagrasib [70, 71]. BI-1823911 was also found to be a synergist with a pan-KRAS SOS1 inhibitor BI-1701963. This combination utilizes the ability of BI-1701963 to shift the balance of KRAS^G12C^ to its GDP-loaded form which is the state BI-1823911 can covalently bind to. While preclinical and clinical data suggests that monotherapy of KRAS^G12C^ inhibitors do not generate sustained responses, BI-1823911 has shown promising combinational synergy with the SOS1 inhibitor BI-1701963 in both in vitro and in vivo studies [71]. It is important to note that BI-1701963 has been tested in combination with adagrasib, but the trial was terminated due to toxicity issues (NCT04975256) [72]. Currently, BI-1823911 is being evaluated in a phase I trial (NCT04973163) in which its safety, pharmacokinetics, and efficacy will be determined both as a monotherapy and in combination with the pan-KRAS SOS1 inhibitor BI-1701963.

#### JNJ-74699157

Developed by Johnson & Johnson, JNJ-74699157 is an orally available selective covalent inhibitor of KRAS^G12C^ advanced cancers [73]. It was explored in a phase 1 clinical trial (NCT04006301) including doses at 100 and 200 mg, however, enrollment was stopped at 10 patients due to dose-limiting skeletal muscle toxicities and a lack of efficacy. JNJ-74699157 was therefore deemed unfavorable for continued development [73].

## Resistance mechanisms to direct inhibitors of KRAS^G12C^

Like any other drug treatment, KRAS^G12C^ inhibitors are subject to varying levels of intrinsic resistance or acquired resistance following extended periods of treatment [77]. In particular, resistance mechanisms against G12C inhibitors are quite complex and heterogeneous with a high variance in co-occurring mutations that can either bypass the inhibitors or reduce their binding effectiveness [58].

### ***Primary resistance to KRAS***^***G12C***^*** inhibitors***

The presence of many cell proliferation and survival pathways presents multiple avenues of resistance to a targeted therapy. For one, mutant cells naturally have extensive levels of variability in their dependence on KRAS for proliferation, including some cell lines that can operate almost independently of KRAS [77, 78]. In both KRAS dependent and independent cell lines, a mutation leading to the activation of alternative pathways such as PI3K-AKT-mTOR, or any downstream gain-of-function mutations in the RAF-MEK-MAPK pathway can lead to significantly diminished KRAS inhibition [11, 79–81]. Activation of these pathways may sustain cancer growth even without gain-of-function mutations, and their activation has been shown to reduce the response to KRAS inhibition [77, 78, 82]. Moreover, KRAS itself may remain active under inhibition due to the presence of secondary mutations that disrupt GTPase activity or promote guanine nucleotide exchange [36, 83]. Treating KRAS^G12C^ mutant H358 or HEK293 with ARS853 resulted in a > 95% reduction in GTP bound KRAS, however, this figure dropped when there is loss-of-function mutation in GTPase (e.g. A59G, Q61L, and Y64A) or gain-of-function mutation in guanine nucleotide exchange (e.g. Y40A, N116H, and A156V) [36]. Even if G12C is the only oncogenic mutation present in the cell, upstream signaling molecules like EGFR or downstream aurora kinase A (AURKA) can stimulate production of new KRAS which, due to their high GTP affinity, will rapidly bind to GTP and activate downstream pathways, bypassing the inhibited KRAS [16, 84, 85].

Other concomitant mutations, especially those of STK11, KEAP1 and TP53, as well as PD-L1 status were hypothesized to potentially affect the response to KRAS^G12C^ inhibitors. Although mutations of STK11 and KEAP1 were found to be negatively associated with the response to KRAS^G12C^ inhibitors in clinical trials, no clear association was observed with TP53 mutation or PD-L1 expression level [44, 86]. Interestingly, in a preclinical study using sgRNA library screening and knockout tumor xenograft models, only the loss-of-function mutation of KEAP1 (but not STK11/LKB1) induced partial resistance to adagrasib [54]. Although KEAP1 mutation induced changes in several hallmark gene signatures (e.g. reactive oxygen species pathway, MTORC1, and KRAS signaling), more mechanistic studies are needed. In addition, whether similar findings can be observed in patients’ tumor tissue is ready to be explored [54]. Based on these data, studies are needed to further clarify the importance of patient stratification based on mutation status. In fact, one cohort in KRYSTAL-1 trial was to investigate the response to adagrasib in NSCLC patients with concomitant KRAS^G12C^ and STK11 mutations (NCT03785249).

### ***Acquired resistance to KRAS***^***G12C***^*** inhibitors***

While undergoing treatment, much of the same intrinsic resistance mechanisms can be acquired after an initial response to treatment. For example, in the CodeBreaK100 clinical trial for sotorasib (NCT03600883; Table 3), 28% of NSCLC patients undergoing treatment acquired at least one new genetic alteration [87]. When discussing these types of mutations, there are generally two categories that they fall under: on-target and off-target [77]. On-target resistance that prevents inhibitors from binding typically arose as a result of acquired secondary KRAS mutations disrupting noncovalent binding or amplification of the KRAS^G12C^ allele [53]. In vitro exploration of this resistance used both sotorasib and adagrasib and used 142 resistant Ba/F3 clones, of which, 124 (87%) were found to have 12 different secondary KRAS mutations (Y96D/S, G13D, R68M/S, A59S/T, Q99L, V8E, M72I, Q61L, and I24L) with varying conferred resistance levels [80]. Of the identified mutations, resistance index (RI) was used as a measurement of impaired inhibition relative to G12C alone (RI = 1). Particularly effective resistance mechanisms (RI > 100) on sotorasib alone included G13D, A59S/T, and R68M while Q99L was only highly resistant to adagrasib. Of more importance, however, was the extremely high potency of Y96D/S against both adagrasib and sotorasib binding [80]. Clinically, 17 of 38 patients (45%) explored by Awad et al. with G12C mutant cancers exhibited some form of acquired resistance and among those, the secondary KRAS mutations (Y96C, G12D/R/V/W, G13D, Q61H, R68S, H95D/Q/Q) were found in 4 patients and resulted in resistance to adagrasib [53]. In another similar observation of a G12C treatment with adagrasib, Tanaka et al., discovered 3 secondary KRAS mutations as well (G12D/V and Y96D). In terms of allele amplification, Priest et al. identified G12C allele frequency increases from 3.7% to 74.2% to 97% as treatment continued in a patient with KRAS^G12C^ and ROS1 fusion undergoing TKI (entrectinib) and G12C inhibitor (sotorasib) treatment [88]. In 2 of the patients in Awad et al.’s study, focal KRAS^G12C^ amplification was identified independently of other resistance mechanisms [53].

**Fig. 1:**
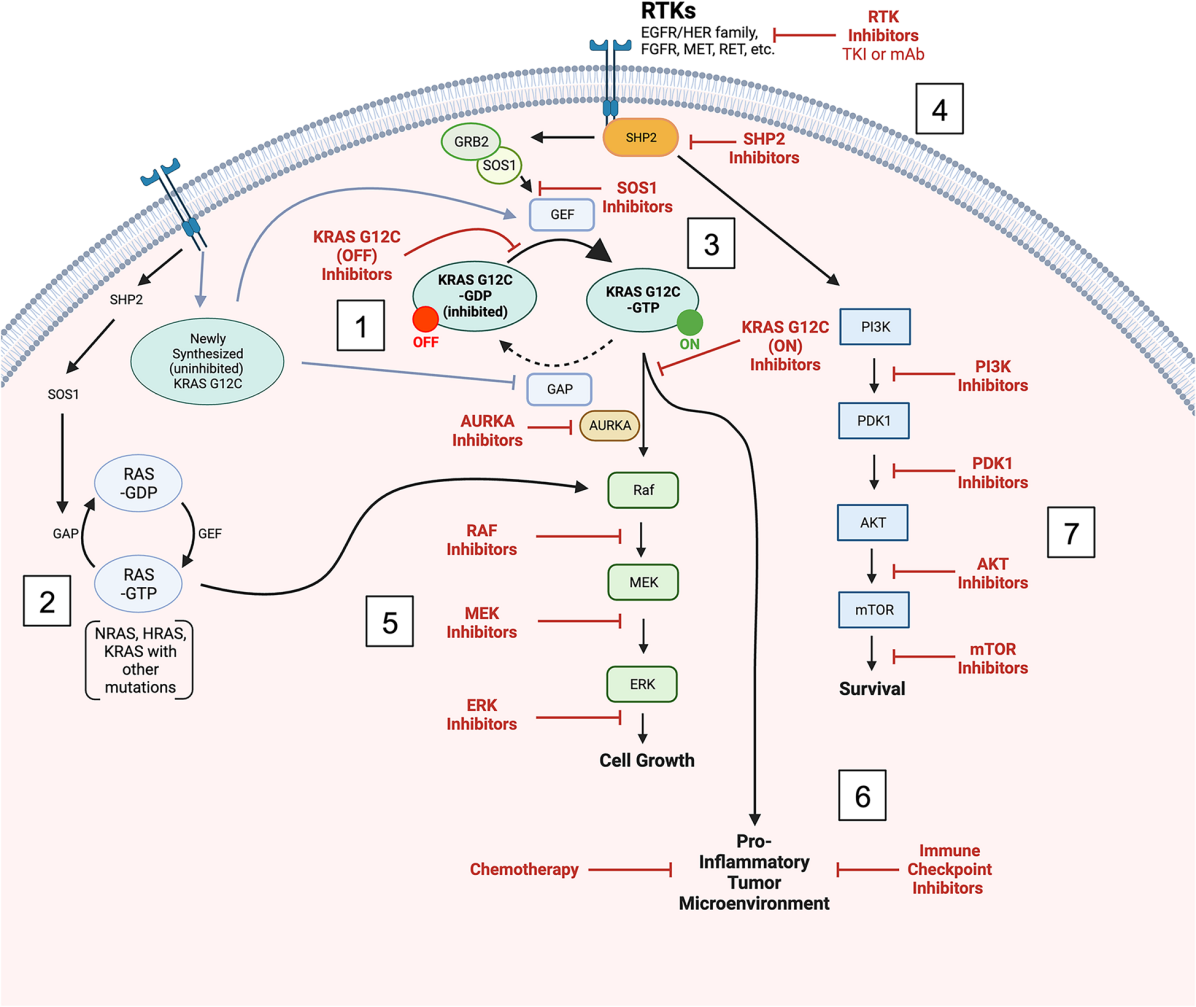
**KRAS**^**G12C**^ **signaling, mechanisms of resistance, and strategies of combating resistance**: **(1)** Inhibitor bound KRAS^G12C^-GDP is unable to be affected by GEF and can’t enter the activated GTP-bound state, however, upstream upregulation of RTKs and KRAS production can overcome the inhibition by stimulating GEF to act on the newly produced KRAS and inhibiting the function of GAP allowing for a constitutively on KRAS^G12C^-GTP to drive cell growth down the MAPK pathway. **(2)** Mutation or amplification of other RAS isoforms can feed into the MAPK pathway and drive inhibitor resistant growth. **(3) **Direct inhibition of GTP bound KRAS^G12C^ to target upstream or KRAS mediated resistance. **(4)** Upstream inhibition of RTKs (EGFR/HER, FGFR2, MET, RET, etc.), SHP2, or SOS1 can target the mutated/upregulated upstream bypass mechanisms. **(5)** Downstream MAPK pathway inhibition using AURKA, RAF, MEK, and ERK inhibitors can be used to target multiple mechanisms of resistance including (1) and (2). **(6)** KRAS^G12C^ promotes a pro-inflammatory tumor microenvironment which diminishes lasting anti-tumor functionality. Cytotoxic chemotherapeutic agents, cell checkpoint inhibitors, or PD-1/L1 inhibitors can help promote tumor cell death. **(7) **The PI3K/AKT/mTOR pathway is activated in parallel during KRAS^G12C^ inhibition and can be targeted with (4) or via pathway specific PI3K, AKT, or mTOR inhibitors. _Created with BioRender.com_

Off-target resistance describes the ability of cells undergoing inhibitor treatment to bypass the inhibition block and the two main downstream pathways (RAF/MAPK and PI3K/AKT) [77]. The first of these mechanisms includes mutations of WT RAS homologs (HRAS, NRAS, and MRAS) that can signal for downstream activation and proliferation of the RAF/MAPK pathway [89, 90]. Specifically, mutations to HRAS and NRAS were found in low frequency in patients, xenografts, and cell line models exhibiting resistance to sotorasib or adagrasib [53, 58, 90]. In those same studies, both downstream and upstream mutations were found as potential resistance mechanisms. In terms of upstream, KRAS activation relies on signaling that starts with a receptor kinase, continues to effector molecules like SHP2 and SOS1, and results in the nucleotide exchange and activation of KRAS [89]. Activating mutations to EGFR, MET, RET, and FGFR2 as well as a diverse array of RTKs caused upregulation of WT RAS and ultimately, resistance to G12C inhibition [53, 85, 90]. These upstream mutations ultimately lead to parallel activations of the MAPK, PI3K, and JNK pathways [77]. Downstream mutations were similarly identified and involved BRAF, RAF1, MAP2K1, and MYC [53, 58, 90]. Several molecules including EGFR, MET, FGFR2, and MYC were also amplified during inhibition [53, 90]. Awad et al. also classified several oncogenic fusions involving ALK, RET, FGFR3, BRAF, and RAF1 as well as loss-of-function mutations to tumor suppressors such as NF1 and PTEN, which can lead to increased activation of the MAPK and PI3K pathways respectively [53]. Alternatively, more diverse resistance mechanisms involved dysregulation of the cell cycle as a result of alterations to cell cycle regulators (e.g. CDKN2A, RB1, and CDK4/6), an increase in TGF-β signaling which induced coagulation, angiogenesis, and alterations to fatty acid and xenobiotic metabolic pathways, and a reduced adaptive immune cell population in the sotorasib resistant tumors [91]. Overall, this widely heterogeneous cell resistance response need only occur in a small proportion of cells to cause broad clinical resistance during treatment [92] (Fig. 1).

## Approaches to target resistance

Various possible methods of targeting resistance are currently being explored and will be discussed in detail below with regard to their approach, preclinical results, and/or current trials. Primarily, improvements to the direct inhibition itself with several new small molecule inhibitors that can bind more effectively or differently (e.g. using noncovalent binding approach) to different status (ON vs. OFF) of the KRAS molecule can theoretically overcome some of the resistance mechanisms [58]. Combination approaches especially targeting the upstream and downstream of RAS–MAPK pathway to maximize the vertical inhibition [53, 58, 80, 87, 89, 90], as well as targeting PI3K–AKT pathway for parallel inhibition [77] are found to be effective. Finally, as KRAS G12C mutation is involved in a proinflammatory TME, dysregulated cell cycle and autophagy, combinatorial efficacy with immunotherapy, chemotherapy and autophagy inhibition is anticipated [5, 42, 91, 93–95].

### ***Novel inhibitors to counteract the resistance to KRAS***^***G12C***^***(OFF)-inhibitors***

#### RM-018

The identification of the Y96D secondary mutation in the S-IIP demonstrated a need for inhibitors that wouldn’t be affected by the alteration at the previous inhibitor class’s binding site [58]. Particularly, RM-018 from Revolution Medicines is a tri-complex inhibitor that utilizes cyclophilin A (CypA) to target and block GTP-bound KRAS^G12C^ from interacting with downstream molecules regardless of the presence of the Y96D mutation [13, 58, 96–98]. RM-018 showcased potent in vitro and in vivo inhibition of MAPK signaling in both G12C and G12C/Y96D mutant cell lines and xenografts with only ~ twofold IC50 shift [58, 96]. In comparison, use of sotorasib, adagrasib, or ARS-1620 resulted in significantly elevated IC50 shifts (> 100-fold, > 100-fold, and ~ 20-fold respectively) and showcased their inability to bind effectively on the Y96D co-mutated KRAS [58, 96]. RM-018 as a novel therapeutic mechanism is yet to enter clinical trials.

#### RMC-6291

Similar to RM-018, RMC-6291 developed by Revolution Medicines is a tri-complex KRAS^G12C^(ON) inhibitor using CypA that was presented at the AACR in 2022 as a potent potential treatment with a pre-clinical ORR of 72% and DCR of 92% in models with KRAS^G12C^ NSCLC and a potential for reduced resistance during treatment [99]. RMC-6291 is currently recruiting for Phase 1/1b monotherapy dose escalation and expansion study in patients with advanced KRAS^G12C^ mutant solid tumors (NCT05462717).

#### RMC-6236

Also developed by Revolution Medicines is a pan-RAS tri-complex inhibitor that can target KRAS (G12V), NRAS, and HRAS among other mutant RAS isoforms utilizing CypA to bind onto GTP-bound RAS [100]. The inhibitor is currently recruiting for a phase 1/1b clinical trial aimed at evaluating safety and tolerability of RMC-6236 on patients with advanced KRAS^G12C^ mutant solid tumors (NCT05379985). Because of its effectiveness against RAS isoforms, a combination therapy of RMC-6236 with a G12C inhibitor could theoretically prevent resistance via RAS isoform activation and improve treatment results.

#### BI-2852

Developed by Boehringer Ingelheim, BI-2852 can bind to KRAS at nanomolar affinity at a pocket between the Switch I and II regions to inhibit effector molecule binding on both GDP and GTP bound KRAS^G12D^ [89, 101]. If this binding mechanism can be used selectively on G12C, a potential therapeutic agent that can affect both OFF and ON status of KRAS will likely have strong potential to counter resistance mechanisms.

#### BI-2493/BI-2865

Developed by Boehringer Ingelheim with the goal to achieve pan-KRAS inhibition. As it is not clear whether the GDP-bound inactive-state selective trapping mechanism afforded by covalent G12C inhibitors will work against non-G12C KRAS mutants, the covalent warhead was removed from a G12C inhibitor prototype, and further optimized to develop into BI-2865, a non-covalent pan-KRAS inhibitor that was able to dampen tumor growth in multiple KRAS mutation carrying cell lines with similar potency to sotorasib [102]. BI-2493 is an in vivo analog of BI-2865 and was found capable of attenuating tumor growth in mice bearing KRAS G12C, G12D, G12V and A146 mutants. However, it was noted that drug treatment on WT KRAS cells caused upregulation of other RAS isoforms, indicative possible resistance mechanisms against treatment [102].

### Vertical pathway coupled inhibition

Despite efforts to provide an all-in-one resistant treatment for G12C mutant NSCLC, the reality is that the most likely approach for successful treatment will come in the form of combinational therapy. One such mechanism with extensive preclinical support for diminished adaptive resistance includes the use of vertical pathway inhibitors in combination with direct KRAS inhibitors [85]. As previously discussed, several adaptive resistance mechanisms occur upstream of the KRAS mutation [53, 85, 89, 90]. In assessing the efficacy of targeting these upstream compounds, Ryan et al. used cell line panels with monotherapies of ARS-1620 and AMG-510 and identified a wide variety of RTK phosphorylation activity that suggested the necessity of targeting the effector molecule SHP2 a common mechanism among the heterogeneity. Erlotinib (EGFR inhibitor), afatinib (pan-HER inhibitor), and BGJ398 (FGFR inhibitor) showed enhanced anti-tumor effects relative to the monotherapy of ARS-1620, due to their abilities to increase GDP-bound KRAS levels as well as combat negative feedback reactivation of RTKs [85, 103]. However, the lack of consistent effectiveness across all cell lines confirmed that variability would hinder their effectiveness, despite the strong synergy scores (scores greater than 10 using growth inhibition matrices and Loewe additivity excess) with AMG-510 (erlotinib = 12.4 & afatinib = 21.3) [42, 85]. Up to now, various KRAS G12C inhibitors that have recently undergone trial have demonstrated therapeutic synergy with cetuximab along with manageable safety profiles [35, 60, 74].

Thus, research shifted towards targeting effector molecules between the RTKs and KRAS including SOS1 and SHP2 [79]. The SOS1 inhibitor BAY-293 produced a synergistic response with ARS-853 in tumor cell lines [79, 104]. However, the KRYSTAL-14 trial utilizing adagrasib and a different SOS1 inhibitor (BI1701963) was terminated due to toxicity concerns (NCT04975256) [72]. RMC-4550, an SHP2 inhibitor, is currently undergoing several combinational clinical trials after showing strong synergy and significant improvements in anti-tumor capabilities with several direct KRAS^G12C^ inhibitors [96, 105] as well as the highest synergy score (22.8) in NCI-H358 treated with AMG-510 [42]. The SHP2 inhibitor TNO-155 is also being explored in a phase Ib/II study in combination with JDQ-443 for treatment of advanced solid tumors (NCT04699188; Table 3) where together they showed partial response in a patient with NSCLC who had previously received chemotherapy and immunotherapy (carboplatin/pemetrexed/pembrolizumab, docetaxel, tegafur–gimeracil–oteracil, and carboplatin/paclitaxel/atezolizumab) [106].

MEK/ERK inhibition downstream of KRAS has also shown strong synergy with AMG-510 in H358 cells (14.7) and showcased both in vitro and in vivo enhancement of sotorasib efficacy when compared to the monotherapy [42]. Although high level of RAS induction was noted when MEK/ERK were inhibited, it can be prohibited when used in combination with a direct G12C inhibitor [85]. Multiple levels of vertical targeting can be used even further in combination as demonstrated by the successful use of SOS1 inhibitor (BI-3406) and MEK inhibitor (trametinib) on Ba/F3 resistant cells with the Y96D/S mutations [80]. This practice of coupling upstream inhibitors with downstream inhibitors to supplement direct KRAS^G12C^ inhibitors is currently under trial in many varying combinations (Table 3).

### Parallel pathway coupled inhibition

Coupling with parallel pathway inhibitors like PI3K/AKT/mTOR inhibitors has also shown relative success in combating resistance in ARS-1620 resistant patient derived xenografts [107]. By closing off two major pathways contributing to cancer cell proliferation and survival, the coupling of PI3K and G12C inhibitors provides a more broadly effective approach in comparison to coupling with a specific SHP2 or MEK/ERK inhibitor due to comparably better tolerance of PI3K monotherapy in patients with lung cancer as well as the fact that reactivation mechanisms under MEK/ERK inhibition are extremely variable [85, 107]. Moreover, combined inhibition of mTOR and IGF1R enhanced the impact of ARS-1620 both in vitro and in mouse models [40].

### Other combinational approaches

Along with the synergy that KRAS^G12C^ inhibitors exhibit with the aforementioned pathway inhibitors, the prospect of combining KRAS inhibitors with chemotherapy or immunotherapy introduces a more refined first-line treatment approach for NSCLC patients with the KRAS^G12C^ mutation. Use of chemotherapeutics like carboplatin in NCI-H358 cells as a monotherapy yielded inhibited tumor growth, but when used in combination with AMG-510, significant anti-tumor activity was observed instead [42]. The same enhanced anti-tumor activity in combination with chemotherapy was also particularly noted during the in vivo preclinical studies of D-1553 [57].

When discussing immune therapy in patients with NSCLC, it is important to first acknowledge the varying efficacy when used in monotherapy as only a small percentage of NSCLC patients directly benefit from PD-1/PD-L1 checkpoint inhibitors [5]. Interestingly, patients with high PD-L1 expression were found more likely to have a KRAS mutation, and a subset of patients harboring KRAS^G12C^/TP53 co-mutations was reported to be particularly responsive to pembrolizumab [108, 109], suggesting a potential role of immunotherapy in KRAS^G12C^ mutant NSCLC. Moreover, patients with the KRAS^G12C^ mutation are typically subject to an immunosuppressive tumor microenvironment (TME) with a correlated increase in PD-L1 expression, and a decrease in the effectiveness of CD8+ due to downregulation of major histocompatibility complex (MHC) class I [5]. However, CT-26 mice treated with G12C inhibitors presented with a remodeled, proinflammatory TME marked by increased infiltration of CD4+ T cells, CD8+ T cells, CD19+ B cells, NK cells, dendritic cells, and M1-polarized macrophages relative to the non-treated mutant mice [5, 42, 93–95]. Interestingly, the anti-tumor response created during KRAS inhibition treatment only became durable when supplemented with a PD-1 inhibitor [95]. Using CT-26 mice as well, Canon et al. yielded tumor regression in only 1 of 10 mice on an anti-PD-1 monotherapy compared to regression in 9 of 10 mice on a combination of anti-PD-1 and sotorasib therapy who also remained cured for 112 days after treatment stopped [42]. At the human level, using pre- and post-treatment paired tumor tissue, adagrasib was found capable of regulating several pathway gene signatures, particularly upregulation of inflammation and immune-related genes along with decreased Ki67 as well as increased PD-L1 expression and CD8+ tumor infiltrating T cells [54, 55]. In combination with pembrolizumab these findings were supplemented by the development of new T cell clones and further increases in CD8+ populations [54, 55]. In a preliminary report of the KRYSTAL-7 trial (evaluable N = 53), adagrasib and pembrolizumab (a PD-1 inhibitor) showcased an ORR of 49% (NCT04613596; Table 3) [110]. It is important to note that the same trial noted a 9% grade 3+ increase in liver function tests which were consistent with AST/ALT elevation when either drug was used in monotherapy [110]. Liver toxicity was also noted in the CodeBreaK 100/101 trials of sotorasib with atezolizumab or pembrolizumab as the most common grade 3+ TRAE but numerically much higher (~ 49%, NCT03600883, NCT04185883; Table 3) [111]. Combination with CDK4/6 inhibitors is also being explored in a clinical trial with adagrasib given the pre-clinical efficacy in treatment of NSCLC [25].

Lastly, KRAS mutant cells experience increases in constitutive autophagy as a survival mechanism, which is further increased when direct inhibitors of the MAPK pathway are used [112]. Thus, adding an inhibitor of master autophagy regulator such as ULKs could potentiate KRAS inhibition via simultaneous suppression of autophagy. Indeed, DCC-3116 (an ULK inhibitor) in combination with sotorasib demonstrated improved efficacy compared to either drug alone in the preclinical setting [112]. Such combination approach is currently being explored in a phase I/II clinical trial (NCT04892017) (Table 4).

**Table 3:** Combinational therapies undergoing clinical trial for KRAS^G12C^ mutant cancer patients

KRAS^G12C^ inhibitor	Identifier	Phase	Objective	Current Status
AMG-510 Sotorasib (LUMAKRAS)	NCT03600883 (CodeBreaK 100)	1/2	Estimate maximum tolerated dose (MTD) and/or recommended dose for phase 2 regimen (RP2D) followed by safety and tolerability of sotorasib in combination with anti PD-1/L1 and midazolam (GABA-A receptor agonist)	Active, not recruiting
	NCT04185883 (CodeBreaK 101)	1b/2	Evaluate the safety and tolerability of sotorasib in monotherapy and in combination with AMG 404 (PD-1 inhibitor), trametinib (MEK inhibitor), RMC-4630 (SHP2 inhibitor), afatinib (EGFR inhibitor), pembrolizumab (PD-1 inhibitor), panitumumab (EGFR inhibitor), carboplatin/pemetrexed/docetaxel/paclitaxel (chemotherapy), atezolizumab (PD-L1 inhibitor), everolimus (mTORC1 inhibitor), palbociclib (CDK4/6 inhibitor), MVASI (VEGF inhibitor), TNO155 (SHP2 inhibitor), FOLFIRI (chemotherapy), FOLFOX (chemotherapy, and BI 1701963 (SOS1 inhibitor)	Recruiting
	NCT04720976	1/2a	Assess the safety and tolerability and determine RP2D of JAB-3312 (SHP2 inhibitor) in combination with binimetinib (MEK inhibitor), pembrolizumab (PD-1 inhibitor), osimertinib (EGFR inhibitor), and sotorasib	Recruiting
	NCT05480865 (Argonaut)	1a/1b	Dose escalation/expansion and optimization of BBP-398 (SHP2 inhibitor) in combination with sotorasib	Recruiting
	NCT05074810 (RAMP203)	1/2	Safety, tolerability, and efficacy of avutometinib (MEK inhibitor) in combination with sotorasib	Recruiting
	NCT05118854	2	Safety and tolerability of sotorasib in combination with chemotherapy (cisplatin/carboplatin) AND pemetrexed (chemotherapy)	Recruiting
	NCT05180422	1/2	Dose expansion followed by safety and tolerability of sotorasib in combination with MVASI (VEGF inhibitor)	Recruiting
	NCT05374538	1a/1b	Dose escalation of VIC-1911 (AURKA inhibitor) as monotherapy or in combination with sotorasib	Recruiting
	NCT05313009	1b/2	Dose expansion followed by safety and efficacy of sotorasib in combination with tarloxotinib (EGFR inhibitor)	Recruiting
	NCT05638295	2	Testing the use of sotorasib as monotherapy or in combination with panitumumab (EGFR inhibitor) in patients with advanced/metastatic malignant solid KRAS^G12C^ mutant cancers	Not yet recruiting
	NCT04892017	1/2	Dose escalation/expansion of DCC-3116 (ULK1/2 inhibitor) as monotherapy and in combination with sotorasib and other RAS/MAPK inhibitors in patients with advanced or metastatic solid tumors harboring RAS/MAPK pathway mutations	Recruiting
MRTX849 Adagrasib (KRAZATI)	NCT03785249 (KRYSTAL-1)	1/2	Evaluate safety, tolerability, pharmacokinetics, metabolites, pharmacodynamics, and clinical activity of adagrasib in monotherapy and in combination with pembrolizumab (PD-1 inhibitor), cetuximab (EGFR inhibitor)	Recruiting
	NCT04330664 (KRYSTAL-2)	1/2	Dose escalation and expansion followed by safety, tolerability, pharmacokinetics, metabolites, pharmacodynamics, and clinical activity of adagrasib in combination with TNO155 (SHP2 inhibitor)	Active, not recruiting
	NCT04418661	1/2	Dose escalation/expansion followed by safety, tolerance, and efficacy of SAR442720 (SHP2 inhibitor) in combination with adagrasib and pembrolizumab (PD-1 inhibitor)	Active, not recruiting
	NCT04613596 (KRYSTAL-7)	2	Efficacy and safety of adagrasib monotherapy and in combination with pembrolizumab (PD-1 inhibitor)	Recruiting
		3	Compares efficacy of adagrasib and pembrolizumab against pembrolizumab and chemotherapy (cisplatin OR carboplatin)	Recruiting
	NCT04975256 (KRYSTAL-14)	1/1b	Safety, tolerability, pharmacokinetics, metabolites, pharmacodynamics, and clinical activity of BI 1701963 (SOS1 pan-KRAS inhibitor) in combination with adagrasib	Completed
	NCT05178888 (KRYSTAL-16)	1/1b	Evaluate pharmacodynamics and preliminary clinical activity of adagrasib in combination with palbociclib (CDK4/6 inhibitor)	Active, not recruiting
	NCT05375994 (RAMP204)	1/2	Safety, tolerability, and efficacy of avutometinib (MEK inhibitor) in combination with adagrasib	Recruiting
	NCT05472623 (NeoKan)	2	Evaluate clinical safety, feasibility, and efficacy of adagrasib in monotherapy or in combination with nivolumab (PD-1 inhibitor)	Not yet recruiting
	NCT05578092	1/2	Evaluate safety, tolerability, pharmacokinetics, pharmacodynamics, and anti-tumor activity of MRTX0902 (SOS1 inhibitor) as monotherapy or adagrasib	Recruiting
	NCT05609578 (KRYSTAL-17)	2	Clinical efficacy and safety of pembrolizumab (PD-1 inhibitor) in combination with adagrasib	Recruiting
GDC-6036	NCT04449874	1a/1b	Dose escalation/expansion, safety, pharmacokinetics, and preliminary activity of GDC-6036 (KRAS^G12C^ inhibitor) in monotherapy and in combination with atezolizumab (PD-L1 inhibitor), cetuximab (EGFR inhibitor), bevacizumab (VEGF inhibitor), erlotinib (EGFR), GDC-1971 (SHP2 inhibitor, and inavolisib (PI3K inhibitor)	Recruiting
D-1553 (garsorasib)	NCT04585035	1/2	Identify the MTD/RP2D and evaluate the safety and tolerability of D-1553 (KRAS^G12C^ inhibitor) as monotherapy or in combination with other standard treatments of solid tumors, NSCLC, or colorectal cancer (CRC)	Recruiting
	NCT05492045	1b/2	Dose escalation followed by safety, tolerability, pharmacokinetics, and efficacy of D-1553 (KRAS^G12C^ inhibitor) in combination with other targeted therapies or immunotherapy	Not yet recruiting
JDQ443	NCT04699188 (KontRASt-01)	1b/2	Dose escalation/expansion assessing safety, anti-tumor activity, tolerability, pharmacokinetics, and pharmacodynamics of JDQ443 (KRAS^G12C^ inhibitor) in monotherapy or in combination with TNO155 (SHP2 inhibitor) and tislelizumab (PD-1 inhibitor)	Recruiting
	NCT05358249 (KontRASt-03)	1b/2	Dose escalation followed by safety, tolerability, and anti-tumor activity of backbone JDQ443 (KRAS^G12C^ inhibitor) in combination with trametinib (MEK inhibitor), ribociclib (CDK4/6 inhibitor, or cetuximab (EGFR inhibitor)	Recruiting
LYS3537982	NCT04956640	1a/1b	Evaluate safety, tolerability, and preliminary efficacy of LY3537982 (KRAS^G12C^ inhibitor) in combination with abemaciclib (CDK4/6 inhibitor), erlotinib (EGFR (PD-1 inhibitor), temuterkib (ERK inhibitor), LY3295668 (AURKA inhibitor), cetuximab (EGFR inhibitor), and TNO155 (SHP2 inhibitor)	Recruiting
BI 1823911	NCT04973163	1a/1b	Dose escalation/expansion to investigate the safety, pharmacokinetics and preliminary efficacy of BI 1823911 (KRAS^G12C^ inhibitor) in monotherapy or in combination with BI 1701963 (SOS1 inhibitor) and midazolam (GABA-A receptor agonist)	Recruiting
JAB-21822	NCT05002270	1/2	Dose escalation/expansion followed by safety and tolerability of JAB-21822 (KRAS^G12C^ inhibitor) as monotherapy or in combination with cetuximab (EGFR inhibitor)	Recruiting
	NCT05288205	1/2a	Dose escalation/expansion evaluating efficacy and safety of JAB-21822 (KRAS^G12C^ inhibitor) in combination with JAB-3312 (SHP2 inhibitor)	Recruiting
IBI351	NCT05504278	1b/2	Evaluate efficacy and safety of IBI351 (KRAS^G12C^ inhibitor) in combination with sintilimab (PD-1 inhibitor), chemotherapy (pemetrexed and/or cisplatin/carboplatin), and cetuximab (EGFR inhibitor)	Not yet recruiting

**Table 4:** Summary of combinational approaches [25, 35, 40, 42, 57, 60, 71, 79, 85, 103, 104, 107, 112–114]

Combinational pathway	Target	Combinations discussed (Ex./KRAS G12Ci)	Results
Vertical (upstream)	*EGFR*	Cetuximab or erlotinib/LY3537982	Improved tumor regression in H358 xenografts compared to cetuximab monotherapy Clinical Trial: NCT04956640
		Erlotinib/sotorasib	Strong synergy in H358 xenografts marked by score > 10 (12.4)
	*HER*	Afatinib/sotorasib	Strong synergy in H358 xenografts marked by score > 10 (21.3) Clinical Trial: NCT04185883
	*SHP2*	RMC-4550/sotorasib	Strong synergy in H358 xenografts marked by score > 10 (22.8)
		RMC-4550/D-1553	Reduced tumor volume in H358 xenografts compared to either drug individually after 24 days of study
		TNO155/JDQ443	Greater tumor efficacy when used together in NCI-H2030 CDX models Clinical Trial: NCT04699188
	*SOS1*	BAY-293/ARS-853	Synergy identified with combination index significantly below 0.8 (according to median-effect model of Chou-Talalay) demonstrating parallel inhibition
		BI-1701963/adagrasib	KRYSTAL-14: terminated due to toxicity concerns Clinical Trial: NCT04975256
		BI-1701963/BI-1823911	Tumor regression in 9/9 H2122 NSCLC cell models Clinical Trial: NCT04973163
(Downstream)	*AURKA*	LY3295668/ LY3537982	Improved tumor regression in H358 xenografts compared to LY3295668 monotherapy Clinical Trial: NCT04956640
	*MEK/ERK*	Trametinib/sotorasib	Strong synergy in H358 xenografts marked by score > 10 (14.7) CodeBreaK101 phase 1b results demonstrated a DCR of 83.3% in 18 KRAS^G12C^ mutant NSCLC patients (3 received prior G12C inhibitors) Clinical Trial: NCT04185883
		Trametinib/D-1553	Reduced tumor volume in H358 xenografts compared to either drug individually after 24 days of study
Parallel	*PI3K*	GDC0941/ARS-1620	Well tolerated combination and reduced tumor growth compared to either therapy alone in patient-derived cell lines with G12C mutated lung cancer
	*mTOR/IGF1R*	Everolimus/linsitinib/ARS-1620	Profound inhibition of cell viability using all 3 drugs in combination on H358 cells in both 2D and 3D conditions providing evidence of strong, durable reduction of tumor cell growth H358 xenografts were more sensitive to G12C direct inhibitor when combined with IGF1R and mTOR inhibition and achieved more durable regression whereas ARS-1620 monotherapy was ineffective as resistance developed
Chemotherapy		Carboplatin/sotorasib	Significant anti-tumor growth in combination compared to inhibited tumor growth in monotherapy Clinical Trials: NCT04185883 NCT05118854
		Carboplatin/D-1553	Reduced tumor volume in H358 xenografts compared to either drug individually after 24 days of study Clinical Trial: NCT05492045
Immunotherapy	*PD-1 & PD-L1*	Anti-PD-1 (unnamed)/sotorasib	112 days of tumor regression in 9/10 mice compared to 1/10 in anit-PD-1 monotherapy
		Pembrolizumab/adagrasib	KRYSTAL-7: ORR = 49% and 9% increase in grade 3+ liver TRAEs Clinical Trials: NCT04613596 NCT03785249 NCT04418661 NCT05609578
		Atezolizumab or pembrolizumab/sotorasib	CodeBreaK 100/101: liver toxicity noted as most common TRAE (49%) Clinical Trials: NCT03600883 NCT04185883
	*CDK4/6*	Abemaciclib/LY3537982	Improved tumor regression in H358 xenografts compared to abemaciclib monotherapy Clinical Trial: NCT04956640
		Palbociclib/adagrasib	Near-complete inhibition in H2122 and SW1573 cells when used in combination compared to concentration-dependent partial inhibition with adagrasib alone Clinical Trial: NCT05178888
Autophagy	*ULK1/2*	DCC-3116/sotorasib	Improved tumor inhibition compared to sotorasib monotherapy in KRAS^G12C^ NSCLC preclinical models Clinical Trial: NCT04892017

## Summary

Given the recent strides taken in research and development of several KRAS^G12C^ direct inhibitors for the treatment of NSCLC and the successful FDA approval of both adagrasib and sotorasib, the perspective can shift towards understanding how these drugs and others may be used most effectively. Both an adequate understanding of the basis for adaptive and innate resistance as well as the knowledge of novel resistance mediating approaches are required in order to do so. Given the complexity and variability in resistance mechanisms, therapeutic approaches that combine vertical, parallel, or alternate pathway inhibition may prove to be the most successful ways to combat tumor rebound. The lack of clinical data on the subject presents a challenge in identifying the best combination and sequence of treatment, however, this is more so a symptom of the recency of these scientific developments rather than a gap in the field, which will soon be addressed by various ongoing clinical trials. In the era of immunotherapy, combining KRAS^G12C^ inhibitors with anti-PD-1/L1 agents is likely to be extensively investigated, particularly with the aim of achieving long-term survival benefits. Such combinations may also involve limited cycles of chemotherapy based on PD-L1 expression levels. Given the heterogeneity and plasticity of tumor cells, the most promising approach is expected to be personalized regimens tailored to an individual’s molecular and immune profiles, necessitating continual refinement as emerging data become available. Overall, the progress made in only 10 years since the revolutionary structural discovery at Shokat lab reassures the possibility of a highly effective treatment mechanism that can finally conquer KRAS^G12C^ mutant NSCLC and provides a launching point that can potentially manage other RAS mutant cancers.

## Data Availability

Not applicable.
